# Integrative Multi-Omics Reveals Serum Markers of Tuberculosis in Advanced HIV

**DOI:** 10.3389/fimmu.2021.676980

**Published:** 2021-06-08

**Authors:** Sonya Krishnan, Artur T. L. Queiroz, Amita Gupta, Nikhil Gupte, Gregory P. Bisson, Johnstone Kumwenda, Kogieleum Naidoo, Lerato Mohapi, Vidya Mave, Rosie Mngqibisa, Javier R. Lama, Mina C. Hosseinipour, Bruno B. Andrade, Petros C. Karakousis

**Affiliations:** ^1^ Center for Clinical Global Health Education and Center for Tuberculosis Research, Division of Infectious Diseases, Department of Medicine, Johns Hopkins University School of Medicine, Baltimore, MD, United States; ^2^ Instituto Gonçalo Moniz, Fundação Oswaldo Cruz, Salvador, Brazil; ^3^ Multinational Organization Network Sponsoring Translational and Epidemiological Research (MONSTER) Initiative, Salvador, Brazil; ^4^ Byramjee Jeejeebhoy Government Medical College-Johns Hopkins University Clinical Research Site, Pune, India; ^5^ Department of International Health, Johns Hopkins Bloomberg School of Public Health, Baltimore, MD, United States; ^6^ Department of Medicine, Division of Infectious Diseases, Perelman School of Medicine at the University of Pennsylvania, Philadelphia, PA, United States; ^7^ College of Medicine-Johns Hopkins Project, Blantyre, Malawi; ^8^ Centre for the AIDS Programme of Research in South Africa, Nelson R Mandela School of Medicine, College of Health Sciences, University of KwaZulu-Natal, Durban, South Africa; ^9^ South African Medical Research Council-CAPRISA HIV-TB Pathogenesis and Treatment Research Unit, Doris Duke Medical Research Institute, University of KwaZulu-Natal, Durban, South Africa; ^10^ Soweto ACTG CRS, Perinatal HIV Research Unit, University of the Witwatersrand, Johannesburg, South Africa; ^11^ Durban International Clinical Research Site, Enhancing Care Foundation, Durban, South Africa; ^12^ Asociacion Civil Impacta Salud y Educacion, Lima, Peru; ^13^ University of North Carolina Project-Malawi, Lilongwe, Malawi; ^14^ Division of Infectious Diseases, Department of Medicine, University of North Carolina at Chapel Hill School of Medicine, Chapel Hill, NC, United States; ^15^ Curso de Medicina, Faculdade de Tecnologia e Ciências (FTC), Salvador, Brazil; ^16^ Curso de Medicina, Escola Bahiana de Medicina e Saúde Pública (EBMSP), Salvador, Brazil

**Keywords:** tuberculosis, HIV, microRNA, metabolomics, biomarker, multi-omics

## Abstract

Tuberculosis (TB) accounts for disproportionate morbidity and mortality among persons living with HIV (PLWH). Conventional methods of TB diagnosis, including smear microscopy and Xpert MTB/RIF, have lower sensitivity in PLWH. Novel high-throughput approaches, such as miRNAomics and metabolomics, may advance our ability to recognize subclinical and difficult-to-diagnose TB, especially in very advanced HIV. We conducted a case-control study leveraging REMEMBER, a multi-country, open-label randomized controlled trial comparing 4-drug empiric standard TB treatment with isoniazid preventive therapy in PLWH initiating antiretroviral therapy (ART) with CD4 cell counts <50 cells/μL. Twenty-three cases of incident TB were site-matched with 32 controls to identify microRNAs (miRNAs), metabolites, and cytokines/chemokines, associated with the development of newly diagnosed TB in PLWH. Differentially expressed miRNA analysis revealed 11 altered miRNAs with a fold change higher than 1.4 or lower than -1.4 in cases relative to controls (p<0.05). Our analysis revealed no differentially abundant metabolites between cases and controls. We found higher TNFα and IP-10/CXCL10 in cases (p=0.011, p=0.0005), and higher MDC/CCL22 in controls (p=0.0072). A decision-tree algorithm identified gamma-glutamylthreonine and hsa-miR-215-5p as the optimal variables to classify incident TB cases (AUC 0.965; 95% CI 0.925-1.000). hsa-miR-215-5p, which targets genes in the TGF-β signaling pathway, was downregulated in cases. Gamma-glutamylthreonine, a breakdown product of protein catabolism, was less abundant in cases. To our knowledge, this is one of the first uses of a multi-omics approach to identify incident TB in severely immunosuppressed PLWH.

## Introduction

In resource-limited countries, human immunodeficiency virus (HIV) and tuberculosis (TB) account for a large burden of infectious disease and contribute significantly to morbidity and mortality. In 2019, there were an estimated 690,000 deaths from HIV/AIDS, with 33% of deaths attributed to HIV-associated TB (1). HIV increases the risk of reactivation of latent TB 20-fold, with escalating risk as CD4^+^ T cells decline (2). HIV and TB co-infection subsequently becomes a lethal combination, with each infection accelerating the progression of the other, as both lead to deterioration of immunologic function.

There is an urgent need to identify persons living with HIV (PLWH) at risk of developing TB, as these individuals could benefit from enhanced monitoring and clinical assessment. Conventional methods of TB diagnosis have limitations in PLWH, as sputum smear microscopy is negative in 24-61% cases of pulmonary TB and HIV co-infection (3). The rapid molecular assay Xpert MTB/RIF offers enhanced diagnostic capabilities but, for smear-negative cases, has an estimated sensitivity of only 55% in PLWH, compared to 67% in HIV-negative individuals (4, 5). Furthermore, the use of sputum-based diagnostic assays does not adequately address extrapulmonary TB, a more common disease in PLWH (6, 7). Thus, novel rapid molecular assays using other readily available biospecimens are urgently needed to improve the diagnosis of both pulmonary and extrapulmonary TB in HIV-infected individuals.

Increasingly, there has been a shift to using host-based assays for TB diagnosis. *Mycobacterium tuberculosis* infection profoundly alters host metabolism and whole-body energy consumption, and metabolites have been profiled in plasma and serum using ^1^H nuclear magnetic resonance (NMR) spectroscopy and liquid chromatography with tandem mass spectrometry (LC-MS/MS) (8–14). In addition to metabolites, host microRNAs (miRNAs) have been studied as circulating biomarkers for various diseases, including TB (15, 16). miRNAs are stable, small, noncoding RNAs involved in the regulation of gene expression, apoptosis, cell cycle control, and development (17), and their dysregulation has been implicated in the pathogenesis of numerous cancers and autoimmune diseases (18–20), as well as TB and other infectious diseases (21–26).

Previous studies have focused on identifying circulating host metabolite or miRNA profiles for TB diagnosis, however there are limited data on the changes of these analytes in the serum of patients with TB and HIV co-infection. Furthermore, HIV infection alone leads to changes in host serum metabolites and miRNAs (27–33), thus the profile of altered metabolites and miRNAs in TB and HIV co-infection may differ compared to either TB infection or HIV infection. In this study we used a multi-omics approach to identify metabolites, miRNA, and cytokines/chemokines associated with the development of newly diagnosed TB in PLWH, leveraging clinical data and biospecimens from the AIDS Clinical Trials Group Study 5274 “Reducing Early Mortality and Morbidity by Empiric TB Treatment” (REMEMBER) (34). We hypothesize that TB induces changes in the metabolism and inflammatory state of the HIV-infected host which can be detected in the serum and can be used for the diagnosis of pulmonary and extrapulmonary TB. The novel use of a multi-omics approach in HIV/TB co-infection could further identify contributory pathways in the development of TB and could highlight future potential therapeutic targets to aid in the prevention of TB morbidity and mortality.

## Materials and Methods

### Study Design

We conducted a case-control study from participants enrolled in REMEMBER, an international, multi-site, open-label randomized control trial comparing empiric 4-drug TB therapy with isoniazid preventive therapy in PLWH (34). This study assessed TB and mortality in adults with HIV and CD4^+^ T cell counts <50 cells/µL within 48 weeks of initiating antiretroviral therapy (ART).

### Study Population

REMEMBER trial participants were recruited from 18 outpatient research clinics in 10 countries (Malawi, South Africa, Haiti, Kenya, Zambia, India, Brazil, Zimbabwe, Peru, and Uganda) (34). A total of 850 participants were enrolled from October 31, 2011, to June 9, 2014. All participants were HIV-infected, ART-naïve individuals, aged 13 years or older, with a CD4^+^ T cell count <50 cells/µL, and had no evidence of active TB. Participants were randomized to receive empiric 4-drug TB therapy or isoniazid preventive therapy and were all initiated on ART. At baseline, participants were screened for TB prior to enrolling, with all 18 sites using symptoms screening, microscopy for identification of acid-fast bacilli in sputum, sputum culture, chest radiography, and only 5 sites using Xpert MTB/RIF assay. Individuals were excluded if they had confirmed or suspected TB, had received TB therapy within 96 weeks prior to study entry, had received isoniazid preventive therapy 48 weeks prior to study entry, or had a household contact diagnosed with multidrug-resistant TB. Other inclusion criteria included liver transaminase (AST or ALT) levels ≤2.5 times the upper limit of normal, a creatinine clearance of at least 30 mL/min, and a Karnosky score of at least 30.

For our case-control study, we randomly selected 23 cases who developed incident TB, defined as a TB diagnosis within 48 weeks of randomization. Cases were selected from 57 TB events in the parent trial, based on sample availability. The specimen used for biomarker analysis was selected from the scheduled study visit closest to the time of TB diagnosis. Participants originated from 5 clinical sites in South Africa, India, and Peru. Incident TB cases were either microbiologically confirmed or were adjudicated by an external clinical TB endpoint review committee. For each case, up to two study-time and site-matched controls were randomly selected by incidence density sampling, with a total of 32 controls selected. For controls, a stored biospecimen within +/- 4 weeks of the time of the corresponding case TB diagnosis was used for biomarker analysis. Cases and controls were followed for 96 weeks after study entry.

### Sample Collection

Six mL of whole blood was collected in plain vacutainer and was transported to the processing lab at ambient temperature within 2 hours of collection. Blood was allowed to clot up to 30 minutes and was spun at 1000-1200 x g for 10 minutes. Serum aliquots were prepared and stored at -70°C. Each site shipped serum on dry ice to the United States. Per participant, a total of one aliquot of 1mL of serum was used to complete miRNA, metabolite, and cytokine analyses.

### miRNA Next Generation Sequencing (NGS)

RNA was isolated using the miRNeasy Serum/Plasma Advanced Kit (QIAGEN) according to the manufacturer’s recommendation. In brief, library preparation was performed using the QIAseq miRNA Library Kit (QIAGEN). A total of 5 μl RNA was converted into miRNA NGS libraries. Adapters containing unique molecular identifiers (UMIs) were ligated to the RNA. Then RNA was converted to cDNA with amplification of cDNA using PCR followed by sample purification. Library preparation quality control (QC) was performed using either Bioanalyzer 2100 (Agilent) or TapeStation 4200 (Agilent). The libraries were pooled in equimolar ratios and were quantified using qPCR. The library pool was then sequenced on a NextSeq500 sequencing instrument according to the manufacturer instructions. Raw data was de-multiplexed and FASTQ files for each sample were generated using the bcl2fastq software (Illumina, Inc.). FASTQ data were checked using the FastQC tool. Cutadapt (1.11) was used to extract information of adapter and UMI in raw reads, and output from Cutadapt was used to remove adapter sequences and to collapse reads by UMI with in-house script. Bowtie2 (2.2.2) was used for mapping the reads.

### miRNA Statistical Analysis

The count miRNA expression matrix was examined using the DESeq2 package from R 4.0.2 to identify differentially expressed miRNAs following the comparison of cases versus controls based on the metadata (35). We defined miRNA as differentially expressed when statistical test values (False Discovery Rate adjusted p-value) were lower than 0.05 and the fold change/difference was higher than 1.4 or lower than -1.4. A total of 2555 miRNAs were used in the analysis. Candidate differentially expressed miRNAs were visualized in a volcano plot with EnhancedVolcano package from R (version 4.0.2). For the enrichment analysis, the targets from the differentially expressed miRNAs were retrieved from mirTarBase and scanned by the REACTOME database using compareCluster package from R (version 4.0.2) (36, 37). The expression values from miRNA were normalized with variance stabilizing transformation with *varianceStabilizingTransformation* function, without prior information of samples, and were used for downstream analysis with a decision-tree algorithm.

### Quantitative Metabolomics Analysis

Sample preparation was performed at Metabolon, Inc. (Durham, North Carolina) using the automated MicroLab STAR system from Hamilton Company with quality-control analyses performed as previously described (38, 39). Briefly, for quality control purposes, numerous recovery standards were added prior to the first step in the extraction process. Organic solvent was removed by briefly placing samples on a TurboVap^®^ (Zymark). The sample extracts were stored overnight under nitrogen before preparation for analysis by ultrahigh performance liquid chromatography-tandem mass spectroscopy (UPLC-MS/MS). All methods utilized a Waters ACQUITY UPLC and a Thermo Scientific Q-Exactive high resolution/accurate mass spectrometer (MS) interfaced with a heated electrospray ionization (HESI-II) source and Orbitrap mass analyzer operated at 35,000 mass resolution. Sample extract was dried and reconstituted in solvents for optimization of analysis as previously described (40). MS analysis used dynamic exclusion to alternate between MS and data-dependent MS^n^ scans, with scan range covering 70-1000 m/z. Metabolon’s hardware and software were used to extract, peak-identify, and QC-process raw data, as previously described (40). Metabolon libraries of purified standards or recurrent unknown entities were used to identify compounds.

### Metabolite Statistical Analysis

Group comparison analysis was performed with the omu package in R (version 4.0.2) using a nonparametric test (41). The fold-change value for each compound was estimated with the omu_summary function. A total of 621 metabolites were evaluated. Differentially abundant metabolites were defined when statistical test values (False Discovery Rate adjusted p-value) were lower than 0.05 and the fold change was higher than 1 or lower than -1.

### Quantification of Serum Cytokines and Chemokines

Serum samples were thawed from storage at -80°C and were filtered using a Millipore human cytokine/chemokine magnetic bead method. Serum levels of epidermal growth factor (EGF), fibroblast growth factor (FGF-2), eotaxin/CCL11, transforming growth factor-α (TGF-α), granulocyte colony-stimulating factor (GCSF), FMS-like tyrosine kinase 3 ligand (Flt-3L), granulocyte-macrophage colony-stimulating factor (GM-CSF), fractalkine/CX3CL1, interferon-α2 (IFN-α2), interferon-γ (IFN-γ), growth related oncogene (GRO), IL-10, monocyte chemoattractant protein-3 (MCP-3/CCL7), IL-12, macrophage-derived chemokine (MDC/CCL22), IL-13, IL-15, sCD40L, IL-17/CTLA8, IL1Ra, IL-1a, IL-9, IL-1b, IL-2, IL-3, IL-4, IL-5, IL-6, IL-7, IL-8/CXCL8, IP-10/CXCL10, monocyte chemoattractant protein-1 (MCP-1/CCL2), macrophage inflammatory protein 1-α (MIP-1α/CCL3), macrophage inflammatory protein 1-α (MIP-1α/CCL4), tumor necrosis factor-α (TNFα), tumor necrosis factor-α (TNF-α/LTA), and vascular endothelial growth factor (VEGF) were measured using Luminex assays following vendor guidelines and a Luminex 100 apparatus (Luminex, Oosterhout, Netherlands), according to the manufacturer’s instructions.

### Serum Marker Statistical Analysis

The Luminex data (concentrations in pg/ml) were compared across the groups using the Wilcoxon-Mann-Whitney U test, and the results were displayed in box plots.

### Combining “Omics” Data for Biomarker Identification

The variance stabilizing transformation miRNA expression values, the log-transformed metabolite data, and the pg/ml values from the serum markers were combined into one dataset and were used to perform a decision-tree approach for identification of a minimal variable set to best classify the groups. The analysis input included 2555 miRNAs, 621 metabolites, and 37 cytokines/chemokines. The best tree was indicated by output from the analysis using the Complexity Parameter, which maximizes the tree classification accuracy. The machine-learning-based decision-tree algorithm, with 1000 leave one out cross-validations, was applied to identify the minimal variable (miRNA/metabolite/serum cytokine or chemokine) set which exhibited the highest classification power to describe the cases and controls with the rpart package (42). Principal Component Analysis was performed in R 4.0.2, using the function *prcomp*, in order to compare and visualize grouping in the source data (miRNAomics, metabolomics and cytokines/chemokines). The resulting variables were retrieved from the dataset and the classification was assessed by receiver operating characteristic (ROC) curve and the area under the curve (AUC) values. Subgroup analysis based on microbiological confirmation of TB status was not performed, as specified *a priori*, due to small sample size.

### Ethics Statement

Local ethics committees and the Institutional Review Boards at Johns Hopkins University and participating site institutions approved this study (IRB00123874). Written informed consent was provided by all participants (NCT01380080).

## Results

### Study Population

Among the 23 cases of incident TB, 12 were diagnosed with pulmonary TB (PTB) and 11 were diagnosed with extrapulmonary TB (EPTB). Fourteen cases (61%) were microbiologically confirmed by smear, culture and/or Xpert MTB/RIF assay and the remaining met criteria for diagnosis of TB by an external clinical TB endpoint review committee. The characteristics of cases and controls are shown in **Table 1**. The median time to TB diagnosis in the cases was 4.6 weeks following initiation of ART and TB therapy (either 4-drug empiric therapy or isoniazid preventative therapy) (**Supplemental Figure 1**). The median time from TB diagnosis to specimen collection (occurring during a scheduled clinic visit) was 0.6 weeks. Thirty-one of 32 controls were not suspected of having TB and remained TB-free at up to 96 weeks of observation after study entry. One control had suspected TB meningitis at week 1 but was ultimately diagnosed with cryptococcal meningitis based on the presence of cryptococcal antigen in the cerebrospinal fluid. Repeating analyses excluding this control did not alter the results.

**Table 1:** Characteristics of cases and controls.

Study Characteristics		TB Case (n=23)	Control (n=32)	p-value
Sex (n,%)	Male	13 (56.5)	13 (40.6)	0.41
	Female	10 (43.5)	19 (59.4)	0.41
Age (median, IQR)		34 (31-41)	35 (30.5-41)	0.70
Baseline CD4 (median, IQR)		32 (26-44)	24.5 (14-37)	0.53
Baseline HIV Log Viral Load (median, IQR)		5.69 (5.24-6.22)	5.41 (5.02-5.68)	0.007
WHO Stage 3 or 4 (n,%)		7 (30.87)	7 (21.87)	0.72
TB Therapy Arm (n,%)	Empiric 4-drug	12 (52.17)	16 (50)	0.87
	IPT	11 (47.83)	16 (50)	0.87
Time to TB Diagnosis in Weeks (median, IQR)		4.6 (2-16.1)	—	
Type of TB (n,%)	PTB	12 (52.17)	—	
	EPTB	11 (47.83)	—	
BMI < 18.5 kg/m^2^ (n,%)		6 (26.09)	5 (15.62)	0.67
Albumin (median, IQR)		3.55 (3.1-3.9)	3.8 (3.4-4.3)	0.015
Hemoglobin ≥ 8 μg/dL (n, %)		21 (91.30)	32 (100)	0.09

IQR, Interquartile range; WHO,World Health Organization; TB, Tuberculosis; IPT, Isoniazid preventative therapy; PTB, Pulmonary TB; EPTB, Extrapulmonary TB; BMI, Body Mass Index (BMI).

### Profile of Differentially Expressed miRNAs

**Figure 1:**
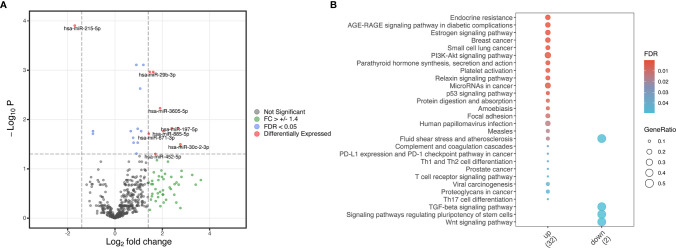
Differentially expressed miRNA in cases versus controls. **(A)** Volcano plot from differentially expressed miRNA identified in cases versus controls based on adjusted p-value and log fold-change of miRNA expression. Red indicates differentially expressed miRNA with both a log fold change (FC) higher than 1.4 or lower than -1.4 and a false discovery ratio (FDR) of lower than 0.05. Green indicates miRNA with a log fold change higher than 1.4 or lower than -1.4, and blue indicates miRNA with a false discovery ratio lower than 0.05. Grey indicates genes without a significant FC or FDR. **(B)** Enrichment analysis plots from differentially expressed genes. The dot sizes represent the gene ratio in the pathway while the fill colors are the FDR values. Only statistically significant enriched pathways are displayed.

Our analysis of differentially expressed miRNAs in serum resulted in 11 altered miRNA with a log-fold change higher than 1.4 or lower than -1.4 in cases relative to controls (p<0.05, **Figure 1A**). Ten miRNAs (hsa-miR-29b-3p, hsa-miR-30c-2-3p, hsa-miR-197-5p, hsa-miR-340-3p, hsa-miR-452-5p, hsa-miR-671-3p, hsa-miR-885-5p, hsa-miR-941, hsa-miR-3127-5p and hsa-miR-3605-5p) were upregulated and one (hsa-miR-215-5p) was downregulated (**Figure 1A**). We performed pathway enrichment analysis of target genes to investigate potential pathways predicted to be influenced by these differentially expressed miRNAs. Twenty-five pathways were found as probably influenced by the upregulated miRNAs and 4 as probably influenced by the downregulated miRNA (**Figure 1B**). Notable pathways targeted by upregulated miRNAs include cell cycle regulation (“PI3K-Akt signaling pathway” and “p53 signaling pathway”), endocrinological pathways, and pathways related to numerous cancers. The TGF-α signaling pathway was influenced by the downregulated miRNA (hsa-miR-215-5p).

### Comparison of Serum Metabolite Levels

Our analysis revealed no differentially abundant metabolites between cases and controls. All differences in serum metabolite abundance were not significant after the False Discovery Ratio (FDR) correction (**Supplemental Figure 2**).

### Comparison of Serum Cytokines and Chemokines

**Figure 2:**
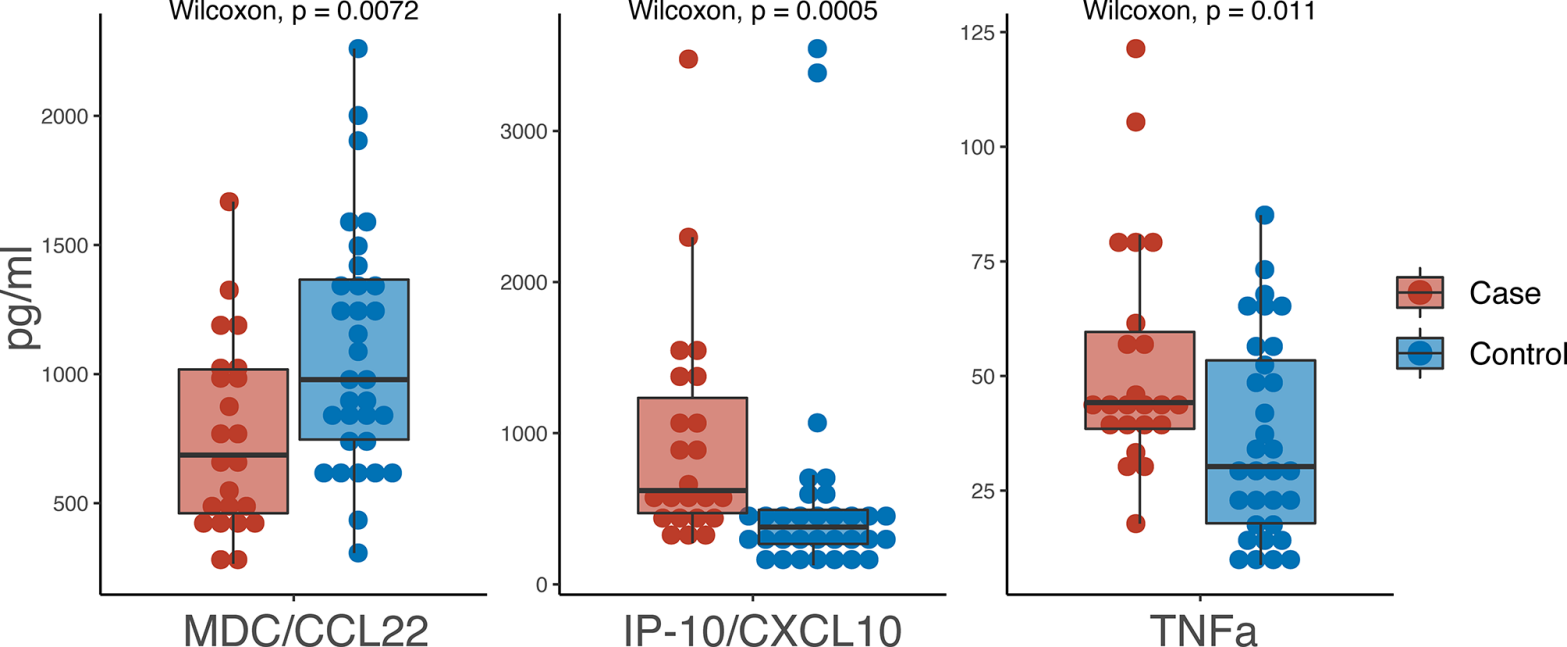
Boxplot of pg/ml values from serum biomarkers. Red indicates cases and blue indicates controls.

Of the 37 cytokines/chemokines measured in serum, we observed 3 with statistically significant differences between cases and controls: TNFα, IP-10/CXCL10 and MDC/CCL22 (**Figure 2**). TNFα was higher in cases (44.2 pg/ml) versus controls (30.25 pg/ml) (p=0.0072) as was IP-10/CXCL10 (619.9 pg/ml in cases versus 378.65 pg/ml in controls; p=0.0005). MDC/CCL22 was higher in controls (978.7 pg/ml) compared to cases (686.2 pg/ml) (p=0.011).

### Combining Omics Data for Identifying a TB Biomarker Panel in HIV Patients

**Figure 3:**
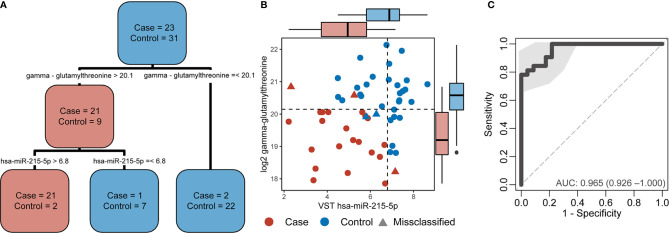
Decision-tree algorithm results applied in the combined multi-omics data. **(A)** Decision-tree from the case and control classification. **(B)** Dot plot from variables selected by the decision-tree with dotted lines the decision thresholds. The boxplots parallel to X-axis show the hsa-miR-215-5p variance stabilizing transformation (VST) values by group and the boxplots parallel to the Y-axis show the log2 gamma-glutamylthreonine values by group. Cases are denoted in red and controls in blue. Circles indicate correctly classified cases and controls whereas triangles indicate misclassifications. **(C)** Receiver operating characteristic (ROC) curve from the decision tree variables demonstrating the sensitivity, specificity, and area under the curve (AUC) of hsa-miR-215-5p and gamma-glutamylthreonine to discriminate participants by TB status.

A decision-tree algorithm identified gamma-glutamylthreonine and hsa-miR-215-5p as the optimal variables to classify incident TB cases (**Figure 3A**). Despite the absence of differentially abundant metabolites in cases versus controls, the log_2_ gamma-glutamylthreonine value was indicated as a classification variable in the decision tree along with variance stabilizing transformation values of hsa-miR-215-5p. Gamma-glutamylthreonine and hsa-miR-215p were less abundant in cases. This metabolite/miRNA pair was able to classify the samples with only 5 errors (**Figure 3B**). Of the 5 misclassifications, two were controls and three were cases. Among the cases, one was cultured-confirmed EBTB, one was non-microbiologically confirmed EPTB, and the last was non-microbiologically confirmed PTB. The metabolite/miRNA pair showed a strong ability to accurately discriminate TB cases from controls with a sensitivity of 0.81 (95% CI 0.66-0.94), a specificity of 0.78 (95% CI 0.61-0.96), and an AUC of 0.965 (95% CI 0.925-1.000) (**Figure 3C**). Integration of cytokine markers did not improve the AUC. Leave-one-out cross validation had an accuracy of 0.907 (95% CI 0.82-0.98), a no-information rate of 0.544, a sensitivity of 0.869, and a specificity of 0.967 with Principal Component Analysis shown in **Supplemental Figure 3**.

## Discussion

In this study, we used three different modalities and integrated omics analysis comparing HIV-infected adults with and without incident TB to identify serum markers characteristic of incident TB. Our case-control study was comprised of severely immunocompromised PLWH initiating ART from geographically diverse regions. Our cases of incident TB developed despite participants receiving either 4-drug empiric TB therapy or isoniazid preventive therapy at the time of ART initiation. We found that 11 miRNAs were differentially expressed in incident TB cases, as were three serum cytokines (TNFa, IP-10/CXCL10 and MDC/CCL22), with significant differences between cases and controls. We found no differentially abundant metabolites between cases and controls at the time of TB diagnosis. Finally, a decision-tree algorithm approach using the multi-omics data revealed that two variables, gamma-glutamylthreonine and hsa-miR-215-5p, had the ability to accurately discriminate incident TB cases from controls with an AUC of 0.965. To our knowledge, this is one of the first uses of a multi-omics approach to identify incident TB in a severely immunosuppressed cohort of PLWH.

Our data contribute to a growing body of literature assessing the role of miRNAs in TB pathogenesis. Only one miRNA, hsa-miR-215-5p, was downregulated in incident TB cases versus controls. hsa-miR-215-5p, a widely studied miRNA found to be dysregulated in numerous cancers, targets genes in the cell cycle and signaling pathways, cell migration, cellular metabolism, and the TGF-β signaling pathway (18). In a case-control study of HIV-negative TB-infected participants, Wang et al. found that miR-215 was significantly increased in patients with TB following two months of treatment, relative to untreated TB patients (25). Our enrichment analysis showed the TGF-α signaling pathway as likely influenced by the downregulated has-miR-215-5p, consistent with previous studies (43, 44). TGF-α has been implicated in TB pathogenesis, through suppression of IFN-γ and with upregulated TGF-α1 inhibiting cytotoxic T-cell function in granulomas, leading to promotion of mycobacterial growth (45, 46).

Some of the pathways targeted by our identified upregulated miRNAs have been linked to TB pathogenesis, including PI3K/AKT/mTORC1 and p53. In a study of individuals with culture-proven pulmonary tuberculosis, *Mycobacterium tuberculosis* (Mtb) was found *in vitro* to inhibit signaling through the PI3K/AKT/mTORC1 pathway, leading to increased MMP-1, thus contributing to a tissue destructive phenotype facilitating granuloma cavitation and TB transmission (47). In an *in vivo* murine model of TB, as well as in human peripheral mononuclear blood cells, pharmacologic inhibition of the AKT/mTOR pathway also led to blunted cellular responses to Mtb (48). Tumor suppressor p53, a regulator of DNA repair, cell cycle arrest, and apoptosis, has also been found to have antituberculosis activity. Mtb has been found to suppress apoptosis in alveolar epithelial cells *in vitro* and this was associated with increased replication of intracellular bacteria (49). Furthermore, macrophages deficient in p53 have higher intracellular survival of Mtb and lower rates of apoptosis compared to wild type macrophages (50). The 10 upregulated miRNAs in our study found to be more abundant in the serum of cases relative to controls have not been associated with TB in prior studies (23–26, 47). The latter studies, however, did not assess the abundance of circulating miRNAs in a severely immunocompromised PLWH cohort, which may account for some of the differences in our findings.

We found that TNFα and IP-10/CXCL10 were elevated in cases at the time of incident TB diagnosis, whereas MDC/CCL22 was elevated in controls. TNFα and IP-10/CXCL10 were recently identified as two biomarkers among a 4-biomarker signature predictive of incident versus prevalent TB in a less immunosuppressed cohort of PLWH (51). IP-10/CXCL10, a chemokine secreted in response to INFγ, has been established as a biomarker of latent and active TB (52–54). IP-10/CXCL10 has also been identified as predictive of incident TB in two additional studies of PLWH (55, 56). High baseline TNFα has recently been found to be associated with incident TB in an HIV-negative cohort (57).

The field of metabolomics has been applied to the study of TB and HIV co-infection, with a recent study finding that precursors of arachidonic acid and linoleic acid metabolism were altered in a TB-IRIS group compared to a non-IRIS group (13). While we did not find any differentially abundant metabolites in our study, gamma-glutamylthreonine, a breakdown product of protein catabolism (58), did have the ability to accurately discriminate incident TB cases from controls when combined with hsa-miR-215-5p (AUC 0.965). A multi-omics approach has been increasingly employed to investigate novel mechanisms of complex diseases, offering insight into genotype-phenotype relationships (59–61).

Our study has several limitations. There is some degree of heterogeneity between cases and controls, given the multi-site nature of the REMEMBER trial and the two treatment arms of empiric 4-drug anti-TB therapy versus isoniazid preventative therapy (34). Furthermore, our highly immunosuppressed cohort of PLWH (CD4^+^ T cell counts <50 cells/μL) had other prevalent and incident co-infections in addition to TB, which likely contributed to further heterogeneity in our results. This could in part explain the lack of differences in serum metabolites between our two groups. Since our study was conducted in participants with advanced HIV, it is unclear if these findings would apply to an earlier stage of HIV.

Another limitation of the study pertains to the selection of controls. Given the case-control nature of the study design, one control was suspected of having TB meningitis but was ultimately diagnosed with antigen-confirmed cryptococcal meningitis. The controls remained TB-free for up to 96 weeks of observation from study entry. Although controls were screened for TB at baseline by symptoms, chest radiography, smear, and sputum culture, some controls received empiric 4-drug anti-TB therapy, which could have treated subclinical TB. However, the effect of such a misclassification would likely have minimized differences between the two groups. Nonetheless, future studies evaluating and validating these markers in participants who did not receive empiric TB therapy would be beneficial. Furthermore, based on sample availability, we had access to a relatively small sample size of cases and controls, limiting our power. We were unable to validate our findings due to limited existing databases containing cytokines, metabolites, and miRNAs studied in a similar cohort of highly immunocompromised PLWH who develop incident TB. Based on the nature of our case-control study design, we were able to evaluate markers at the time of incident TB diagnosis but were not able to extend this to a predictive model, as we did not evaluate serum markers at baseline.

Our findings could provide the basis for future blood-based studies of cytokines, metabolites, and miRNAs for validation and development of a TB diagnostic signature, however further validation is needed, particularly in geographically and ethnically diverse HIV seropositive populations with varying degrees of immune suppression. The WHO Target Product Profile for TB biomarker diagnostic tests recommends development and testing against a gold standard of confirmed pulmonary TB, with a goal specificity of ≥98% and a sensitivity of ≥65% (62). While our model had a sensitivity of 0.81 (95% CI: 0.65-0.93) at a specificity of 98%, it was developed in both confirmed and adjudicated cases of PTB and EPTB. Future directions would include testing this miRNA/metabolite pair in a larger sample of PLWH with culture-confirmed pulmonary TB. In the future, integrated omics analysis could be used in longitudinal cohorts to determine if this miRNA/metabolite pair (or other profiles) is predictive of TB progression in a severely immunocompromised HIV cohort, a group that is at high risk of developing TB and experiencing subsequent mortality due to TB.

In summary, our data indicate that two variables, gamma-glutamylthreonine and hsa-miR-215-5p, had the ability to accurately discriminate incident TB cases from controls in a severely immunosuppressed PLWH cohort. These data provide insight into dysregulated disease pathways in individuals with advanced HIV who developed active TB disease, despite receipt of TB prophylaxis at the initiation of ART.

## Data Availability Statement

The datasets presented in this study can be found in online repositories. The names of the repository/repositories and accession number(s) can be found below: https://www.ncbi.nlm.nih.gov/geo/, GSE166557.

## Ethics Statement

The studies involving human participants were reviewed and approved by Johns Hopkins Institutional Review Board. The patients/participants provided their written informed consent to participate in this study.

## Author Contributions

AG and PK contributed to conception and design of the study. PK, AG, GB, JK, KN, LM, VM, RM, JL, and MH contributed to acquisition of data. AG, NG, AQ, and SK organized the database. AQ, BA, NG, and SK performed statistical analysis and interpreted data. SK wrote the first draft of the manuscript. AQ, AG, BA, and PK wrote sections of the manuscript. All authors contributed to the article and approved the submitted version.

## Funding

Research reported in this publication was supported by the National Institute of Allergy and Infectious Diseases of the National Institutes of Health under award numbers UM1 AI068634, UM1 AI068636, and UM1 AI106701. The work was also supported by the Johns Hopkins Baltimore-Washington-India Clinical Trials Unit (BWI CTU) (NIH/NIAID UM1AI069465). This research was supported by grants from the AIDS Clinical Trials Group (ACTG) and CRDF Global to PK. This work was also supported by the Johns Hopkins University Center for AIDS Research (P30AI094189). SK was also supported by the National Institute of Health T32 AI007291-27. PCK was also supported by the NIH/NIAID grant K24AI143447.

## Disclaimer

The content is solely the responsibility of the authors and does not necessarily represent the official views of the National Institutes of Health.

## Conflict of Interest

The authors declare that the research was conducted in the absence of any commercial or financial relationships that could be construed as a potential conflict of interest.

The handling editor declared a past co-authorship with one of the authors NK.

## Supplementary Material

Supplementary Figure 1 Time to TB diagnosis from study enrollment in cases.

Supplementary Figure 2 Differentially abundant metabolites in cases versus controls. Analysis revealed no differentially abundant metabolites, as differences between cases and controls were not statistically significant after the False Discovery Ratio (FDR) correction.

Supplementary Figure 3 Principal component analysis of different input data. **(A)** miRNA expression data; **(B)** Metabolite data; **(C)** Cytokine/chemokine data.
