# Supplementary figures and images for: Integrative Multi-Omics Reveals Serum Markers of Tuberculosis in Advanced HIV
Source: Front Immunol. 2021 Jun 8;12:676980. doi: 10.3389/fimmu.2021.676980 (PMC8217878; doi:10.3389/fimmu.2021.676980)

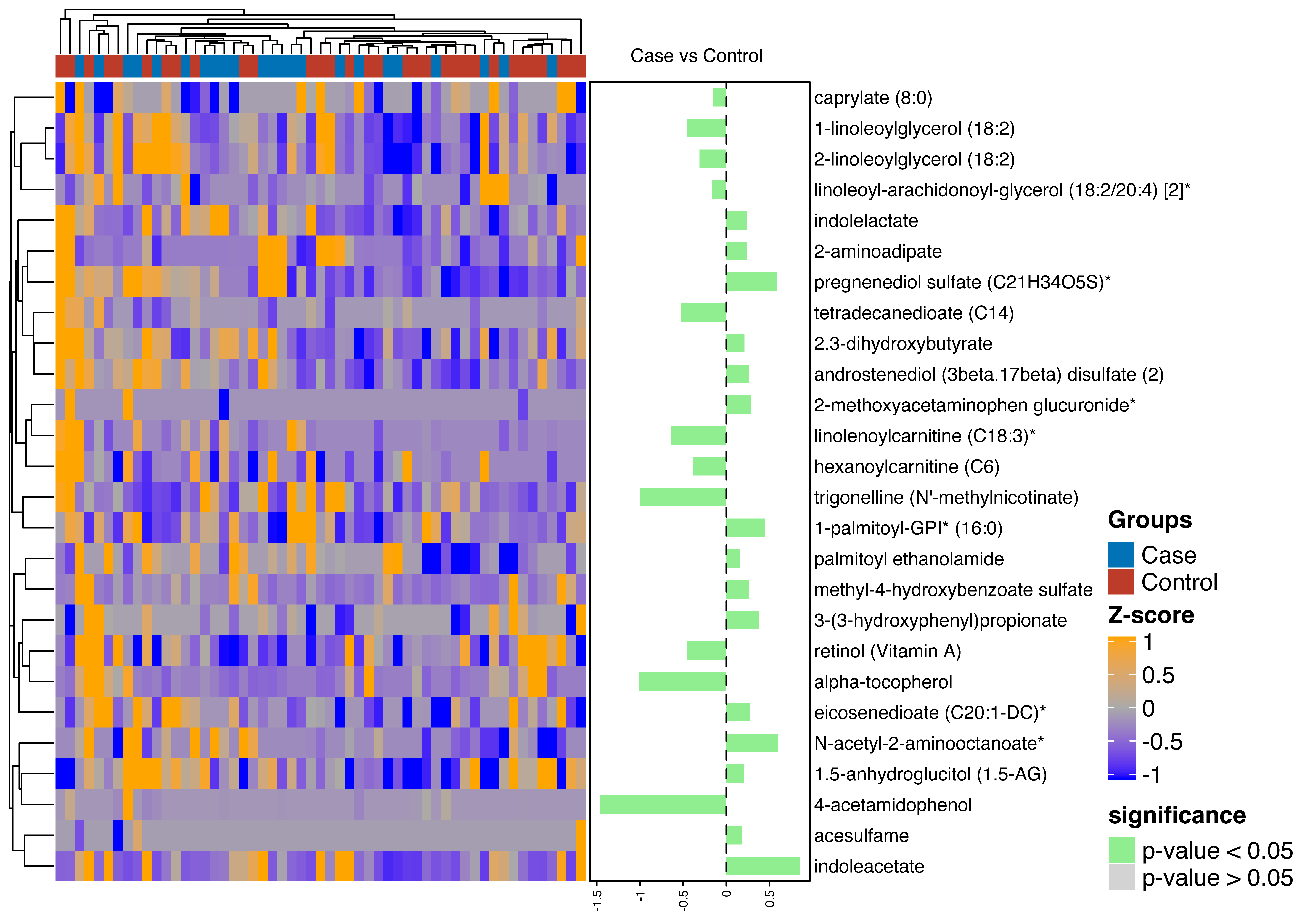

Supplement: Supplementary Figure 2 — Differentially abundant metabolites in cases versus controls. Analysis revealed no differentially abundant metabolites, as differences between cases and controls were not statistically significant after the False Discovery Ratio (FDR) correction. [file Image_2.tif]

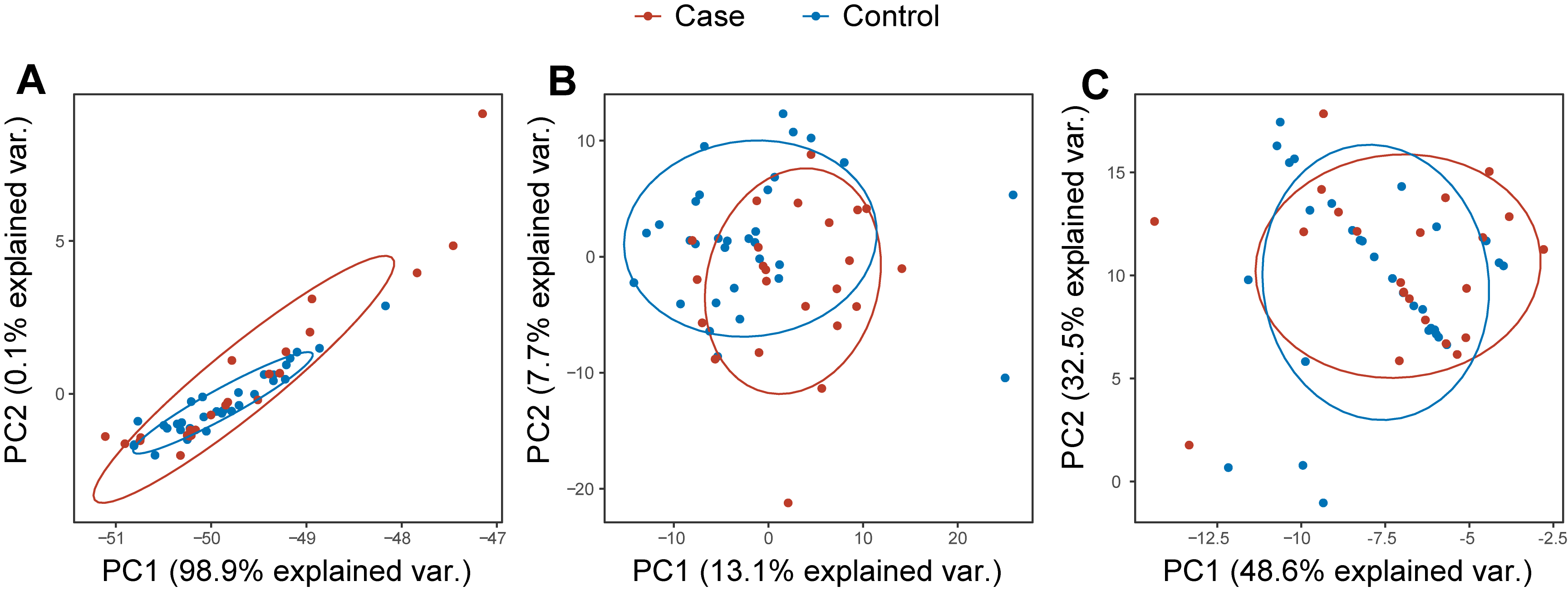

Supplement: Supplementary Figure 3 — Principal component analysis of different input data. (A) miRNA expression data; (B) Metabolite data; (C) Cytokine/chemokine data. [file Image_3.tif]
